# *Andrographis paniculata* Extract and Andrographolide Modulate the Hepatic Drug Metabolism System and Plasma Tolbutamide Concentrations in Rats

**DOI:** 10.1155/2013/982689

**Published:** 2013-08-12

**Authors:** Haw-Wen Chen, Chin-Shiu Huang, Pei-Fen Liu, Chien-Chun Li, Chiung-Tong Chen, Cheng-Tzu Liu, Jia-Rong Chiang, Hsien-Tsung Yao, Chong-Kuei Lii

**Affiliations:** ^1^Department of Nutrition, China Medical University, Taichung 404, Taiwan; ^2^Department of Health and Nutrition Biotechnology, Asia University, Taichung 413, Taiwan; ^3^Department of Nutrition, Chung Shan Medical University, Taichung 408, Taiwan; ^4^Institute of Biotechnology and Pharmaceutical Research, National Health Research Institutes, Miaoli 350, Taiwan

## Abstract

*Andrographolide* is the most abundant terpenoid of *A. paniculata* which is used in the treatment of diabetes. In this study, we investigated the effects of *A. paniculata* extract (APE) and andrographolide on the expression of drug-metabolizing enzymes in rat liver and determined whether modulation of these enzymes changed the pharmacokinetics of tolbutamide. Rats were intragastrically dosed with 2 g/kg/day APE or 50 mg/kg/day andrographolide for 5 days before a dose of 20 mg/kg tolbutamide was given. APE and andrographolide reduced the AUC_0–12 h_ of tolbutamide by 37% and 18%, respectively, compared with that in controls. The protein and mRNA levels and enzyme activities of CYP2C6/11, CYP1A1/2, and CYP3A1/2 were increased by APE and andrographolide. To evaluate whether APE or andrographolide affected the hypoglycemic action of tolbutamide, high-fat diet-induced obese mice were used and treated in the same manner as the rats. APE and andrographolide increased CYP2C6/11 expression and decreased plasma tolbutamide levels. In a glucose tolerance test, however, the hypoglycemic effect of tolbutamide was not changed by APE or andrographolide. These results suggest that APE and andrographolide accelerate the metabolism rate of tolbutamide through increased expression and activity of drug-metabolizing enzymes. APE and andrographolide, however, do not impair the hypoglycemic effect of tolbutamide.

## 1. Introduction

*Andrographis paniculata* (Burm. f) is a medicinal herb cultivated in Southeast Asia. It is widely used as a traditional medicine in Taiwan, China, India, and Thailand for the treatment of infections, cold, fever, inflammation, and diarrhea [1]. Andrographolide is the major diterpene lactone of *A. paniculata* and accounts for 1.7% and 0.8% of the weight of dried leaves and stems of the herb, respectively [2]. *A. paniculata* and andrographolide have recently attracted considerable interest because of their diverse physiological functions and therapeutic potential, including antioxidant [3], anti-inflammatory [4], antiapoptosis [5], antiatherosclerosis [6], anticancer [7], antivirus [8], and hypoglycemia effects [9].

Andrographolide has been shown to suppress tumor necrosis factor *α*-induced intercellular adhesion molecule 1 expression in vascular endothelial cells and to lead to an inhibition of monocyte adhesion to endothelial cells [10]. Animal studies have indicated that the aqueous extract of *A. paniculata,* the ethanolic extract of *A. paniculata* (APE), and andrographolide lower blood glucose levels and induce glucose transporter 4 activity in streptozotocin-induced diabetic rats [11–13]. In a clinical study, andrographolide was reported to inhibit human immunodeficiency virus- (HIV-) induced cell cycle dysregulation and to increase CD4+ lymphocytes in HIV-1-infected patients [14].

 Drug metabolism plays an important role in protecting all living organisms from environmental toxicant insult. Drug metabolism is carried out through Phase I and Phase II drug-metabolizing enzyme systems as well as membrane transporters. Cytochrome P450 (CYP) enzymes are the most important Phase I enzymes responsible for catalyzing the biotransformation of drugs, xenobiotics, and endogenous compounds. In mammals, at least 14 gene families of CYP have been identified [15]. Phase II system comprises the conjugating enzymes that catalyze the conjugation of small water-soluble molecules to both exogenous and endogenous substrates to facilitate their excretion. Uridine diphosphate-glucuronosyltransferases (UGT), glutathione *S*-transferases (GST), and sulfotransferases are three representative Phase II enzymes that detoxify a wide variety of electrophilic xenobiotics by catalyzing their conjugation to glucuronic acid, glutathione (GSH), and sulfate, respectively. Thereafter, conjugates are transported out of cells via Phase III ATP-binding cassette membrane transporter P-glycoproteins [16].

 The gene transcription and activity of drug-metabolizing enzymes and transporters are susceptible to modulation by various dietary nutrient and nonnutrient components, including vitamin E, fatty acids, and phytochemicals such as garlic organosulfur compounds [17, 18]. *A. paniculata* and andrographolide have been reported to be potent modulators of the expression and activity of CYP enzymes. For example, mouse hepatic CYP1A1/2 and CYP3A4 expression and activity are upregulated by treatment with an aqueous extract of *A. paniculata* or APE and with andrographolide [19–21]. However, the effects of *A. paniculata* and andrographolide on CYP gene transcription and enzyme activity remain controversial. In an *in vivo* study, Pekthong and colleagues [22] indicated that CYP2C11 activity and protein expression in rat liver are inhibited by treating rats with a single, daily 5 mg/kg dose of andrographolide or with a single, daily 0.5 g/kg dose of the aqueous extract of *A. paniculata *for 3 days. Inhibition of CYP1A2, CYP2C9, CYP2D6, and CYP3A4 activity and expression by andrographolide was also reported in cultured human and rat primary hepatocytes and HepG2 hepatoma cells [22, 23].

In the case of Phase II conjugating enzymes, mouse liver and kidney GST activity was reported to be significantly increased by the administration of a daily 50 mg/kg dose of the aqueous extract of *A. paniculata* for 14 days [24]. In addition, our previous study showed that andrographolide and APE upregulate the mRNA and protein expression of the *π* form of GST in cultured rat primary hepatocytes [25]. The effects of *A. paniculata* and andrographolide on the expression of UGT, sulfotransferases, and P-glycoproteins have not been investigated.

In the present study, we investigated the modulatory potency of APE and andrographolide on the expression and activity of CYP isozymes, GST, UGT, and P-glycoprotein in rat liver. The effects of APE and andrographolide on tolbutamide pharmacokinetics in rats and on the hypoglycemic effect of tolbutamide in high-fat diet-induced obese mice were also investigated.

## 2. Materials and Methods

### 2.1. Materials

Andrographolide, tolbutamide, cytochrome *c*, dextromethorphan, diclofenac (sodium salt), testosterone, ethoxyresorufin, methoxyresorufin, *p*-nitrophenol, lauric acid, NADPH, GSH, pyrogallol, and 1-chloro-2,4-dinitrobenzene were obtained from Sigma (St. Louis, MO, USA). 4-Hydroxydiclofenac and 6-*β*-hydroxytestosterone were purchased from Ultrafine Chemicals (Manchester, UK). The antibodies raised against rat CYP isozymes were from Chemicon International (Temecula, CA, USA). The antibody against rat P-glycoprotein was from Calbiochem (Darmstadt, Germany). Fresh whole plants of *A. paniculata* were procured from Hualien, Taiwan. All other chemicals and reagents were of analytical grade and were obtained commercially.

### 2.2. Preparation of the Ethanolic Extract of *A. paniculata* (APE)

**Figure 1:**
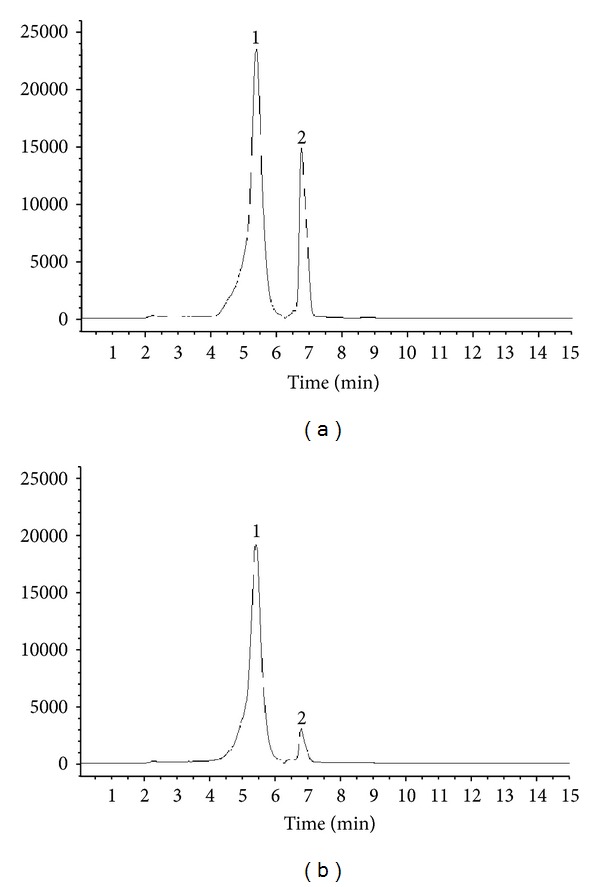
HPLC chromatograms of the andrographolide (1) and 14-deoxy-11,12-didehydroandrographolide (2) standards (1 *μ*g/mL) (a) and the ethanolic extract of* Andrographis paniculata* (APE; 10 *μ*g/mL) (b). Contents of andrographolide (1) and 14-deoxy-11,12-didehydroandrographolide (2) in APE are 53.2 and 30.6 mg/g, respectively.

Ten grams of *A. paniculata* dry powder was extracted with 250 mL of 95% ethanol by stirring overnight at room temperature. The mixture was then centrifuged at 1350 ×g at 4°C for 10 min. The supernatant was concentrated in a rotary evaporator at 55°C under vacuum and was then freeze-dried. The dry powder thus obtained was stored at −20°C. The andrographolide content of APE was determined by LC/MS [26]. APE was found to contain 53 mg of andrographolide and 30 mg of 14-deoxy-11,12-didehydroandrographolide per gram (Figure 1).

### 2.3. Animals and Treatments

Seven-week-old male Sprague-Dawley rats cannulated in the jugular vein were purchased from the Bio LASCO Experimental Animal Center (Taipei, Taiwan). The animals were fed a standard rat diet and were randomly assigned into control, APE-treated, and andrographolide-treated groups (*n* = 5). Rats were housed in plastic cages in a room kept at 23 ± 1°C and 60 ± 5% relative humidity with a 12-hour light and dark cycle. Food and drinking water were available *ad libitum*. Control rats were orally administered methyl cellulose. A dose of 2 g/kg of APE or 50 mg/kg of andrographolide, as a suspension in 0.5% aqueous methyl cellulose (10 mL/kg), was orally administered to each rat daily for 5 days. After the 5th daily dose, rats were deprived of food overnight. On day 6, a 20 mg/kg oral dose of tolbutamide, as a solution in Cremophor EL (polyoxyl 35 hydrogenated castor oil, Sigma)/ethanol/water (30/10/60, v/v/v), was administered to each rat. After the administration of tolbutamide, serial blood samples were collected up to 12 h after dosing from each rat. The animals were then killed by exsanguination, and the livers were collected.

Eleven-month-old high-fat diet-fed (45% of energy from fat; Research Diet Inc., New Brunswick, NJ) C57BL/6 mice were obtained from the National Health Research Institute (Miaoli, Taiwan). Control mice were orally administered 0.5% methyl cellulose (10 mL/kg). Mice were orally administered a 2 g/kg dose of APE or 50 mg/kg of andrographolide, as a suspension in 0.5% aqueous methyl cellulose (10 mL/kg), daily for 5 days (*n* = 6–8). After the 5th daily dose, the mice were deprived of food overnight. On day 6, a 20 mg/kg oral dose of tolbutamide, as a solution in Cremophor EL/ethanol/water (30/10/60, v/v/v), was administered to each mouse. Thirty minutes later, a 1 g/kg dose of glucose, as a solution in water, was administered orally according to the method described by Zong et al. [27]. Serial blood samples were collected up to 8 h after the glucose dose from each mouse. The animals were then killed by exsanguination and the livers collected.

All animals were killed by exsanguination via the abdominal aorta while under carbon dioxide (CO_2_/O_2_, 70%/30%) anesthesia. Heparin was used as the anticoagulant. Plasma was separated from the blood by centrifugation (1750 ×g) at 4°C for 20 min. The liver from each animal was excised, weighed, and stored at −80°C. This research has received ethical approval from the institutional animal ethics committee of China Medical University, Taichung, Taiwan.

### 2.4. Pharmacokinetic Study

 Tolbutamide was prepared in a solution of Cremophor EL/ethanol/water (30/10/60, v/v/v). After administration, blood samples (~200 *μ*L) were collected from the tail vein of each rat at 0.25, 0.5, 1, 2, 4, 8, and 12 h after dosing. Plasma was separated from blood by centrifugation (4000 ×g for 20 min at 4°C). An aliquot of 50 *μ*L plasma was mixed with 100 *μ*L acetonitrile and then centrifuged at 10,000 ×g for 15 min at 4°C. The supernatant was used to determine the concentration of tolbutamide by LC/MS. Calibration standards of tolbutamide were prepared by serial dilution of a tolbutamide stock solution with blank plasma, yielding final concentrations of tolbutamide ranging from 1 to 300 *μ*g/mL of plasma.

The HPLC system consisted of an Agilent 1100 series LC System (Palo Alto, CA) and an Agilent Zorbax Eclipse XDB-C8 column (3 *μ*m, 3.0 mm × 150 mm), interfaced to an Agilent MSD mass spectrometer equipped with an electrospray ionization source. Column temperature was set at 25°C. The initial mobile phase composition was 30% acetonitrile containing 10 mM ammonium acetate (solvent A) and 70% of 0.1% formic acid (solvent B). The following gradient system was used: 30% A to 50% A (0-1 min), 50% A to 98% A (1–3 min), 98% A (3–9 min), 98% A to 30% A (9-10 min), and 30% A (10–15 min). The flow rate was 0.5 mL/min. Sample injection volume was 10 *μ*L. The MS data acquisition was via selected ion monitoring. The positive ion of the testing compound was selected, and the peak size was measured. The area under the plasma tolbutamide concentration versus time curve (AUC) was calculated by using the trapezoidal method.

### 2.5. Determination of Drug-Metabolizing Enzyme Activities

Liver was homogenized (1 : 4, w/v) in an ice-cold potassium phosphate buffer (pH 7.4) containing 7.4 mM K_2_HPO_4_, 2.6 mM KH_2_PO_4_, 1.15% KCl, and 1 mM phenylmethylsulfonyl fluoride (PMSF). Tissue homogenates were centrifuged at 10,000 ×g for 30 min at 4°C. The resulting supernatant was then centrifuged at 105,000 ×g for 1 h at 4°C. The microsomes thus obtained were washed several times to get rid of possible tolbutamide contamination and this can avoid tolbutamide interference of CYP activity, and the microsomes were then suspended in potassium phosphate buffer (pH 7.7) containing 7.5 mM K_2_HPO_4_, 14.5 mM KH_2_PO_4_, and 1 mM EDTA. Ethoxyresorufin (2 *μ*M), methoxyresorufin (5 *μ*M), diclofenac (4 *μ*M), dextromethorphan (60 *μ*M), *p*-nitrophenol (50 *μ*M), testosterone (60 *μ*M), midazolam (2.5 *μ*M), and lauric acid (100 *μ*M) were, respectively, used as the probe substrates for ethoxyresorufin *O*-deethylation (CYP1A1), methoxyresorufin *O*-demethylation (CYP1A2), diclofenac 4-hydroxylation (CYP2C), dextromethorphan *O*-deethylation (CYP2D), *p*-nitrophenol 6-hydroxylation (CYP2E1), testosterone 6*β*-hydroxylation (CYP3A), midazolam 1-hydroxylation (CYP3A), and lauric acid 12-hydroxylation (CYP4A). The microsomal protein concentration used in the enzyme activity assay was 0.2 mg/mL, and the incubation time was 15 min. Metabolites of each CYP enzyme reaction were determined by LC/MS as reported previously [28]. The contents of total CYP and cytochrome b5 were quantified by the method of Omura and Sato [29]. Enzyme activity is expressed as picomoles of metabolite formed per minute per milligram of protein. The NADPH-CYP reductase activity was measured as described by Phillips and Langdon [30] with the use of cytochrome *c* as the substrate. The microsomal protein concentration was determined by using a Coomassie Plus protein assay kit (Pierce, Rockford, IL, USA).

The microsomal UGT activity was determined by using *p*-nitrophenol as the substrate. The rate of formation of *p*-nitrophenol glucuronic acid was measured by HPLC/UV [31]. GST activity was determined spectrophotometrically by measuring the formation rate of GSH conjugates [32].

### 2.6. SDS-PAGE and Western Blotting

Aliquots of microsomal protein (10 *μ*g) were electrophoresed in an SDS-polyacrylamide gel, and proteins were then transferred to polyvinylidene fluoride membranes. After blocking, membranes were hybridized with antibodies to CYP1A1, CYP1A2, CYP2C6, CYP2C11, CYP2D1, CYP2E1, CYP3A1, and CYP3A2.

For P-glycoprotein expression, liver tissue was homogenized with phosphate-buffered saline containing 0.2% Triton X-100, 5 mM EDTA, and 1 mM PMSF. The homogenates were then centrifuged at 10,000 ×g for 30 min at 4°C. Proteins in the resulting supernatants were electrophoretically separated and were immunoblotted with P-glycoprotein antibody. The immunoreactive bands were detected by using an enhanced chemiluminescence plus Western blotting detection reagent (Amersham Biosciences, Boston, MA, USA).

### 2.7. RNA Isolation and RT-PCR

Total RNA in liver samples was extracted by using the Trizol reagent (1 : 20, w/v). RNA (0.4 *μ*g) was reversely transcribed by Moloney murine leukemia virus reverse transcriptase (Promega) in the presence of 150 *μ*M of each dNTP, 40 units RNase inhibitor, and 250 nmol oligo (dT) in a final volume of 20 *μ*L. cDNA was amplified in a thermocycler in a reaction volume of 50 *μ*L containing 20 *μ*L of cDNA, BioTaq PCR buffer, 50 *μ*mol of each dNTP, 1.25 mM MgCl_2_, and 1 unit of BioTaq DNA polymerase (BioLine). The sequences for the PCR primers were as follows: for CYP1A1 (forward: 5′-CCATGACCAGGAACTATGGG-3′; reverse: 5′-TCTGGTGAGCATCCAGGACA-3′), CYP1A2 (forward: 5′-GTCACCTCAGGGAATGCTGTG-3′; reverse: 5′-GTTGACAATCTTCTCCTGAGG-3′), CYP2C6 (forward: 5′-ATAAGACGCTTTACCCTCAC-3′; reverse: 5′-GATTTTCCTGCTTCCACTTA-3′), CYP2C11 (forward: 5′-AGCTCTTGTTGATCTAGGAG-3′; reverse: 5′-GGGAAGTAATCAATAATGGC-3′), CYP2D1 (forward: 5′-TGCCATACAGCCTCTACAAGC-3′; reverse: 5′-CTTGGAAGACCTTGTCAGCC-3′), CYP2E1 (forward: 5′-CTCCTCGTCATATCCATCTG-3′; reverse: 5′-GCAGCCAATCAGAAATGTGG-3′), CYP3A1 (forward: 5′-TTGCCATCACGGACACACAGAAAT-3′; reverse: 5′-GGAGCCACTGGACATTGAGT-3′), CYP3A2 (forward: 5′-AGTAGTGACGATTCCAACATAT-3′; reverse: 5′-TCAGAGGTATCTGTGTTTCCT-3′), and P-glycoprotein (forward: 5′-TTAATGTTTGTATTAATATATGACAC-3′; reverse: 5′-CCATAGACTAAGTTTAAAGGCT-3′). The PCR for CYP1A1 was performed as follows: 5 min at 95°C and then 35 cycles of 30 s at 94°C, 30 s at 54°C, and 60 s at 72°C. For CYP1A2 and CYP3A2 amplification, the PCR cycle number was 30 times through a 30 s denaturing step at 94°C, a 30 s annealing step at 55°C, and a 30 s elongation step at 72°C. For CYP2D1 and CYP3A1, the PCR cycle number was 30 times through a 60 s denaturing step at 94°C, a 60 s annealing step at 56°C, and a 125 s elongation step at 72°C. For CYP2C6, CYP2C11, and CYP2E1, the PCR cycle number was 30 times through a 45 s denaturing step at 94°C, a 25 s annealing step at 57°C, and a 60 s elongation step at 72°C. The PCR amplicons were then electrophoresed in 1% agarose gels containing 40 mM Tris/20 mM glacial acetic acid/2 mM EDTA buffer, and the relative densities of the PCR products were quantitated by use of Image Gauge software (Fujifilm, Tokyo, Japan). The glyceraldehyde 3-phosphate dehydrogenase cDNA level was used as the internal standard.

### 2.8. Extraction of Nuclear Proteins and Electrophoretic Mobility Shift Assay (EMSA)

Nuclear proteins of liver tissues were extracted as described by Tian et al. [33] with some modifications. Briefly, 0.1 g of liver was homogenized (1 : 9, w/v) with 0.9 mL of ice-cold 10 mM HEPES buffer (pH 7.9) containing 10 mM KCl, 1 mM MgCl_2_, 0.1 mM EDTA, 0.5 mM dithiothreitol, 0.5 mM PMSF, 4 *μ*g/mL leupeptin, and 20 *μ*g/mL aprotinin. The homogenates were placed in an ice bath for 15 min and then centrifuged at 600 ×g for 10 min. Then, 100 *μ*L of 10% Nonidet P-40 was added to the supernatant, and the samples were allowed to sit in an ice bath for 10 min. The supernatant was centrifuged at 5,000 ×g for 5 min. The pellet, containing crude nuclei, was resuspended in 100 *μ*L of hypertonic buffer containing 10 mM HEPES, 400 mM KCl, 1 mM MgCl_2_, 0.1 mM EDTA, 0.5 mM dithiothreitol, 4 *μ*g/mL leupeptin, 20 *μ*g/mL aprotinin, 25% glycerol, and 0.5 mM PMSF at 4°C for 45 min. Nuclear proteins were then collected by centrifugation at 12,000 ×g for 15 min.

EMSA was performed according to the method reported previously [34]. The LightShift Chemiluminescent EMSA Kit (Pierce, Rockford, IL) and synthetic biotin-labeled double-stranded consensus oligonucleotides of DR4 (5′-AGCTTCAGGTCACAGGAGGTCAGAGAG-3′) and xenobiotic response element (XRE) (forward: 5′-GATCCGGAGTTGCGTGAGAAGAGCCA-3′) were used to measure pregnane X receptor (PXR) and aryl hydrocarbon receptor (AhR) nuclear protein-DNA-binding activity. Unlabeled double-stranded DR4 and XRE oligonucleotide and a mutant double-stranded oligonucleotide were used to confirm the specificity of protein binding.

### 2.9. Biochemical Assays

Plasma glucose was determined by use of enzymatic kits purchased from Audit Diagnostics Co. (Cork, Ireland). Plasma aspartate aminotransferase (AST) and alanine aminotransferase (ALT) activities were measured by using commercial kits purchased from Randox Laboratories (Antrim, UK).

### 2.10. Statistical Analysis

 Statistical analysis was performed with SAS statistical software (Cary, NC). The significance of the difference among group means was determined by one-way ANOVA followed by Tukey's test; *P *values < 0.05 were taken to be statistically significant. 

## 3. Results

### 3.1. Effects of APE and Andrographolide on Rat Growth

 After 5 days of daily dosing of rats with APE or andrographolide, no significant differences in body weight, liver weight, or relative liver weight were observed between the control and treated rats with the exception of the body weight of the APE-treated rats, which was lower than that of the control and andrographolide-treated rats (*P* < 0.05). Although body weight was affected, liver function parameters, that is, plasma AST and ALT activity, in the APE-fed group were not significantly different from those in the control and andrographolide-treated rats (Table 1).

### 3.2. Effects of APE and Andrographolide on Plasma Tolbutamide Concentrations

**Figure 2:**
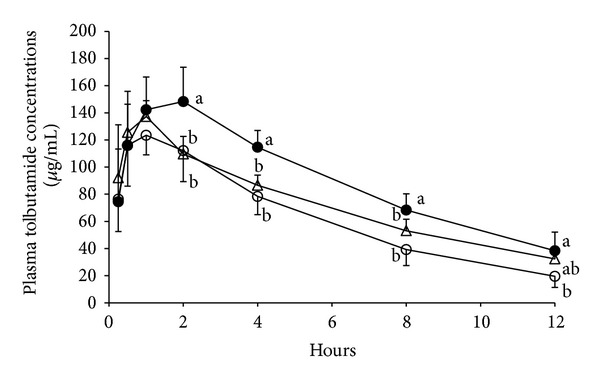
Plasma concentration of tolbutamide after a 20 mg/kg oral dose of tolbutamide to control, ethanolic extract of* Andrographis paniculata-* (APE-) pretreated, and andrographolide-pretreated rats. Rats were given daily a 2 g/kg dose of APE (○) or 50 mg/kg andrographolide (△) for 5 days before the tolbutamide was given. Control rats received 10 mL/kg of 0.5% aqueous methyl cellulose daily (vehicle, *⚫*) for 5 days. Values shown are the mean ± SD, *n* = 5. The significance of the difference among group means was determined by one-way ANOVA followed by Tukey's test. Values of treatments not sharing the same letter differ significantly, *P* < 0.05.

Figure 2 shows the changes in the plasma concentration of tolbutamide after oral administration of a 20 mg/kg dose of tolbutamide to the control, APE-pretreated, and andrographolide-pretreated rats.  Table 2 summarizes the pharmacokinetic parameters of tolbutamide in rat plasma. Lower plasma concentrations of tolbutamide were observed in rats pretreated with APE or andrographolide, which led to a 37% and 18% lower AUC_0–12 h_ in the APE-pretreated (881 ± 152 *μ*g/mL × h, *P* < 0.05) and andrographolide-pretreated (1143 *μ*g/mL × h, 0.1 < *P* < 0.05) rats, respectively, compared with the controls (1393 ± 262 *μ*g/mL × h). 

### 3.3. Effects of APE and Andrographolide on the Activity of Drug-Metabolizing Enzymes

The activities of Phase I and Phase II drug-metabolizing enzymes in the livers of control, APE-treated, and andrographolide-treated rats were presented in Table 3. Compared with that in the control group, total CYP content was higher in the liver of rats treated with APE or andrographolide (*P* < 0.05). Activities of ethoxyresorufin *O*-deethylation (CYP1A1), methoxyresorufin *O*-demethylation (CYP1A2), diclofenac 4-hydroxylation (CYP2C), *p*-nitrophenol 6-hydroxylation (CYP2E1), and testosterone 6*β*-hydroxylation (CYP3A) were also higher in the livers of APE-treated and andrographolide-treated rats than in those of control rats (*P* < 0.05). The UGT activity in the livers of APE-treated rats was also significantly increased to 201% of the control value (*P* < 0.05). APE and andrographolide treatment had no significant effect on dextromethorphan *O*-deethylase (CYP2D), lauric acid 12-hydroxylase (CYP4A), or GST activities in rats.

### 3.4. Effects of APE and Andrographolide on the Induction of Hepatic Drug-Metabolizing Enzymes

**Figure 3:**
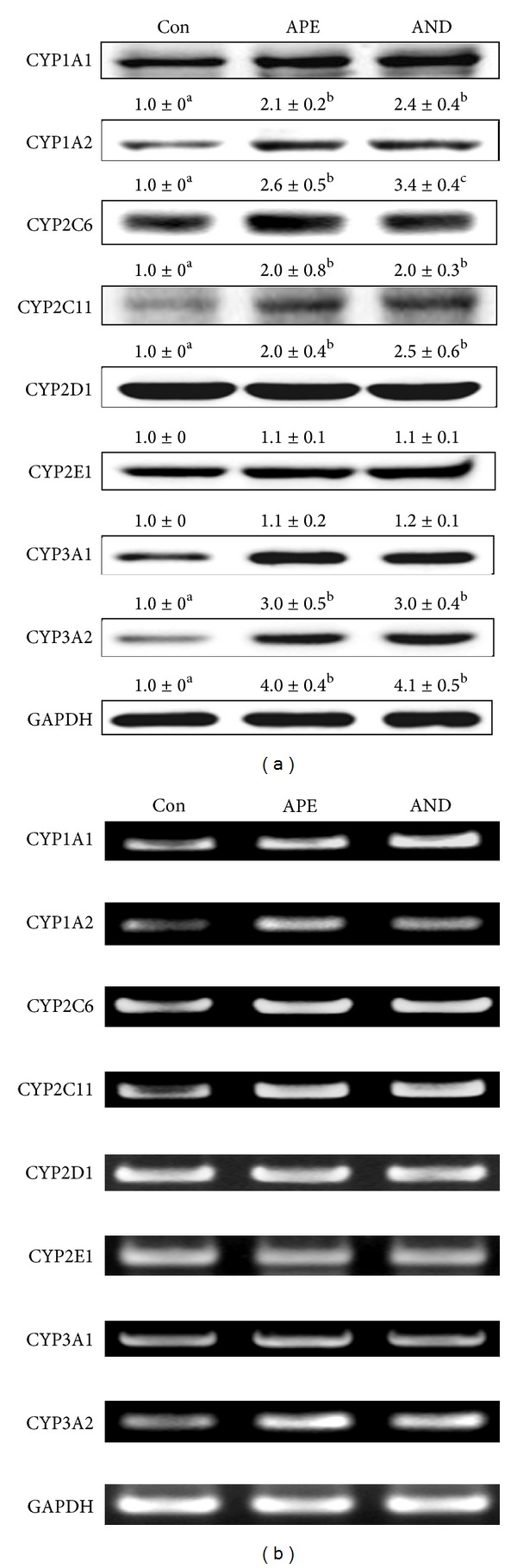
Modulation by ethanolic extract of* Andrographis paniculata* (APE) and andrographolide (AND) of hepatic CYP isozyme protein (a) and mRNA (b) expression. Rats were orally administered methyl cellulose (Con), APE, or AND for 5 days. Total RNA and microsomal proteins were prepared as described in Section 2. Protein and mRNA levels in pooled liver samples (*n* = 5) are shown. Values shown are the mean ± SD, *n* = 5. The significance of the difference among group means was determined by one-way ANOVA followed by Tukey's test. Values of treatments not sharing the same letter differ significantly, *P* < 0.05.

**Figure 4:**
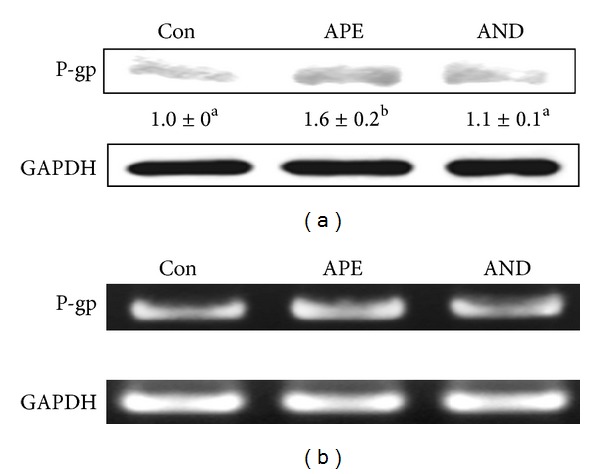
Induction by ethanolic extract of* Andrographis paniculata* (APE) and andrographolide (AND) of the hepatic protein (a) and mRNA (b) expression of P-glycoprotein (P-gp). Rats were orally administered methyl cellulose (Con), APE, or AND for 5 days. Protein and mRNA levels in pooled liver samples (*n* = 5) are shown. Values shown are the mean ± SD, *n* = 5. The significance of the difference among group means was determined by one-way ANOVA followed by Tukey's test. Values of treatments not sharing the same letter differ significantly, *P* < 0.05.

Immunoblots revealed that, consistent with the changes in CYP isozyme activities, CYP1A1, CYP1A2, CYP2C6, CYP2C11, CYP3A1, and CYP3A2 protein levels in rat liver were upregulated by APE and andrographolide (Figure 3(a)). Compared with that in the controls, there was a 1- to 3-fold increase in the protein expression of these CYP enzymes (*P* < 0.05). Similarly, RT-PCR showed that the mRNA levels of CYP1A1, CYP1A2, CYP2C6, CYP2C11, and CYP3A2 were also induced by APE and andrographolide (Figure 3(b)). Although APE and andrographolide were found to increase *p*-nitrophenol 6-hydroxylation, a reaction catalyzed by CYP2E1, there were no changes in CYP2E1 protein or mRNA levels in the liver of APE- and andrographolide-treated rats compared with controls. Increases of protein (Figure 4(a)) and mRNA (Figure 4(b)) levels of membrane transporter P-glycoprotein were observed only in APE-treated rats (*P* < 0.05).

**Figure 5:**
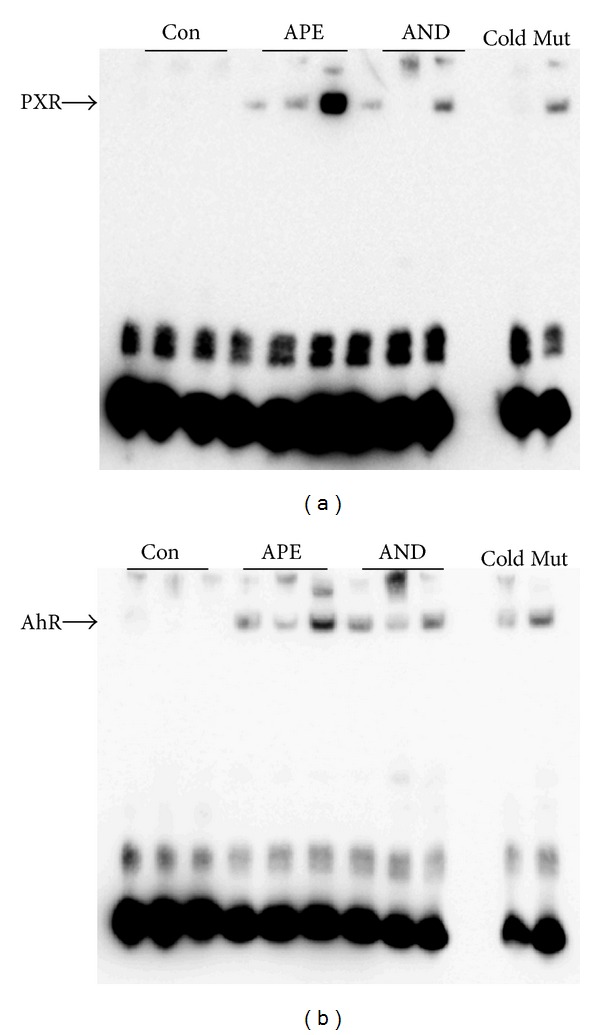
DNA-binding activity of nuclear pregnane X receptor (PXR) (a) and aryl hydrocarbon receptor (AhR) (b). Rats were orally administered methyl cellulose (Con), ethanolic extract of* Andrographis paniculata* (APE), or andrographolide (AND) for 5 days. Nuclear protein extraction and EMSA were performed as described in Section 2. Results of three rats in each group are shown.

PXR and AhR are two important factors in modulating CYP isozyme gene transcription. To confirm the activation of these two proteins in rat liver by APE and andrographolide, EMSA was performed. As shown in Figure 5, both APE and andrographolide enhanced the binding activity of the DR4 and XRE consensus sequences to the nuclear proteins PXR (Figure 5(a)) and AhR (Figure 5(b)), respectively.

### 3.5. Effects of APE and Andrographolide on the Hypoglycemic Effect of Tolbutamide

**Figure 6:**
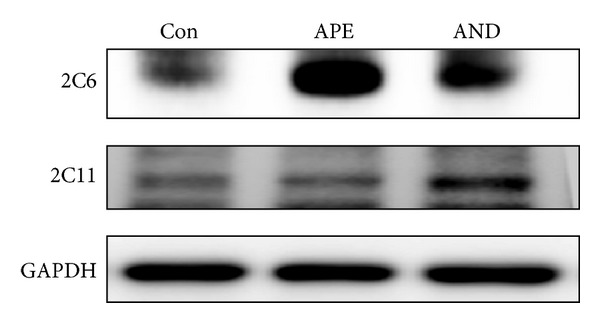
Induction by ethanolic extract of* Andrographis paniculata* (APE) and andrographolide (AND) of hepatic CYP2C6 and CYP2C11 protein expression in high-fat diet-induced obese mice. The animals were orally administered methyl cellulose (Con), 2 g/kg of APE, or 50 mg/kg of AND daily for 5 days. Protein levels in pooled liver samples (*n* = 6–8) are shown.

To evaluate whether the blood glucose-lowering effect of tolbutamide was affected by treatment with APE and andrographolide, a glucose tolerance test was conducted in high-fat diet-induced obese mice. Similar to the changes observed in rats as described above, the hepatic expression of CYP2C6/11, the main CYP enzyme responsible for tolbutamide metabolism, was induced by APE and andrographolide (Figure 6). Furthermore, plasma tolbutamide concentrations, determined at 2 h after dosing, in the APE-treated and andrographolide-treated mice were lower than the plasma tolbutamide concentration of the control mice (29 ± 6 *μ*g/mL, 54 ± 7 *μ*g/mL, and 124 ± 32 *μ*g/mL, resp.). Although treatment with APE and andrographolide reduced the blood tolbutamide level, no increase in the blood glucose level was observed in these animals (Table 4). In contrast, the areas under the plasma glucose concentration versus time curve (AUC_0–8 h_) of the APE-treated (168 ± 50 mg/dL × h) and andrographolide-treated (171 ± 42 mg/dL × h) mice were even lower than that of the control mice (224 ± 87 mg/dL × h). On the other hand, the fasting blood glucose levels in the APE-treated (77 ± 9 mg/dL) and andrographolide-treated (80 ± 7 mg/dL) mice were also lower than that of the control mice (104 ± 18 mg/dL) (*P* < 0.05). These findings suggest that, even though hepatic CYP2C6 and 2C11 expressions are induced, APE and andrographolide do not seem to interfere with the blood glucose-lowering effect of tolbutamide.

## 4. Discussion

Numerous phytochemicals derived from herbs, vegetables, and fruits have attracted much attention in recent years because of their potent and diversified biological functions. Modulation of the activity of drug-metabolizing enzymes is most pronounced in the phytochemical prevention of chemical-induced tissue damage and carcinogenesis [35, 36]. Membrane transporters are also the targets of phytochemicals, which results in changes in drug absorption and efflux [16, 37, 38]. Owing to the critical role of these enzymes in drug metabolism, the possibility exists that herb and phytochemical supplementation could change the metabolic rate of xenobiotics, including therapeutic drugs. In this study, data are presented to demonstrate that the ethanolic extract of *A. paniculata *and its major constituent andrographolide are potent up-regulators of the expression and activities of CYP isozymes and P-glycoprotein and of activity of UGT. This induction of enzyme activity in turn modifies the pharmacokinetics of tolbutamide by decreasing its disposition.

Drug-metabolizing enzymes and membrane transporters are responsible for the elimination of xenobiotics and many endogenous lipophilic. To adapt to this critical role, the gene expression of these enzymes and transporters is susceptible to be modulated by many exogenous compounds, including phytochemicals [18]. A number of nuclear receptors, including PXR, constitute androstane receptor (CAR), peroxisome proliferator-activated receptor *α*, retinoid X receptor, and AhR, and transcriptional factors, including nuclear factor-erythroid 2-related factor 2 (Nrf2) and activating protein 1 (AP-1), have been shown to play an important role in the transcription of genes encoding drug-metabolizing enzymes and transporters [39]. For instance, activation of Nrf2, AhR, PXR, and CAR upregulates the transcription of UGT and GST in a coordinated manner [40, 41]. AhR is the key mediator of 2,3,7,8-tetrachlorodibenzo-p-dioxin-induced changes in CYP1A genes [42]. In the presence of pregnanes, glucocorticoids, and rifampicin, PXR translocates into nuclei, heterodimerizes with retinoid X receptor, binds to the response element as either a direct repeat of the half-site TGAACT spaced by three (DR3) or four (DR4) base pairs or an everted repeat of the TGAACT half-site spaced by six base pairs (ER6), and thereby activates the expression of PXR-driven genes, including CYP3A, CYP2B, CYP2C, UGT, and P-glycoprotein [43, 44].

Phytochemicals are potent activators of the transcription factors stated above. Diallyl sulfide, an organic sulfur compound derived from garlic, increases rat and mouse CYP2B and mouse CYP1A9 and UGT_1A6_ mRNA levels in association with CAR activation [45–47]. Indole and its major metabolite indole-3-carbinol act as a CYP1A1/2 inducer via an AhR-Arnt pathway [48, 49]. *N*-Methylcytisine, a major active component of the Chinese herb medicine* Sophora flavescens*, induces CYP3A4 expression in HepaG2 cells in a PXR-dependent manner [50].

In this study, EMSA clearly revealed that the DNA-binding activity of both PXR and AhR is increased in rats treated with APE and andrographolide (Figure 5). This result suggests that the constituents of APE, at least the andrographolide, act as the activator of PXR and AhR. A computational docking analysis showed that andrographolide binds to AhR with the ligand posing binding energy similar to beta-naphthoflavone (−10.77 versus −9.96 kcal/mol) [51]. Moreover, coincident changes in the DNA-binding activity of AhR and PXR and the mRNA and protein levels of CYP1A1/2, CYP2C6/11, and CYP3A2 in the present study (Figures 3(a) and 3(b)) suggest that APE and andrographolide increase the activity of ethoxyresorufin *O*-deethylation, methoxyresorufin *O*-demethylation, diclofenac 4-hydroxylation, and testosterone 6*β*-hydroxylation (Table 3) in rat liver at the transcriptional level. Unexpectedly, CYP3A1 mRNA did not correlate with CYP3A1 protein in this study (Figure 3). Western blot of CYP3A1 reflects the net result of translation and posttranslational regulation. As reported by Eliasson et al. [52], the cellular CYP3A1 level is regulated to a significant extent posttranslationally. The lack of correlation between CYP3A1 mRNA and CYP3A1 protein after APE and AND treatment might be explained via posttranslational regulation of CYP3A1 by APE and AND.

The transcription of genes encoding Phase II conjugation enzymes and Phase III membrane transporters is coregulated as described for Phase I CYP isozymes by a similar signaling network. In the presence of various CYP inducers, such as 2,3,7,8-tetrachlorodibenzo-p-dioxin, phenobarbital, and dexamethasone, induction of P-glycoprotein expression in the intestine and liver tissues is commonly accompanied by an increase in CYP1A1/2, CYP2B, CYP2C, and CYP3A transcripts [53]. In the present study, the coinduction of CYP isozymes and P-glycoprotein expression was seen only in APE-treated rats; andrographolide showed only a minor effect on P-glycoprotein (Figures 4(a) and 4(b)). This discrepancy suggests that activation of PXR and AhR is not necessary for the induction of P-glycoprotein in rat liver. A large body of evidence has indicated that P-glycoprotein gene transcription is mediated by Nrf2, NF*κ*B, and AP-1 [54–56]. Further study is warranted to explore whether APE and andrographolide have differential roles in the activation of Nrf2, NF*κ*B, and AP-1.

Diterpene lactones are recognized to be the most bioactive components of *A. paniculata*, and andrographolide is the major one in terms of abundance and bioactive properties [2]. Recently, the second most abundant diterpenoid, 14-deoxy-11,12-didehydroandrographolide, was also reported to display several biological activities, including immunomodulation, cardiovascular protection, antihepatotoxicity, and anti-infection activity [1]. In addition to andrographolide (which contains 53 mg/g APE), 14-deoxy-11,12-didehydroandrographolide is also present in the APE preparation as shown in Figure 1, and its content is 30.6 mg/g. This suggests that 14-deoxy-11,12-didehydroandrographolide may be involved in the APE induction of P-glycoprotein. In this study, rats dosed with APE had less weight gain than rats dosed with AND (Table 1); plasma aspartate aminotransferase and alanine aminotransferase activities were not changed by APE and AND compared to the controls (data not shown). These results suggested that 2 g/kg APE and 50 mg/kg AND have no deleterious effect on rat liver function, whereas components other than AND in the APE may exert adverse effect on the body weight gain of rats.

Accompanied by the changes in the activity of drug-metabolizing enzymes and transporters induced by APE and andrographolide, the metabolism of coadministered drugs is potently affected, which leads to herb-drug interactions. Tolbutamide, a 1st generation hypoglycemic drug, is primarily oxidized into hydroxytolbutamide via the action of CYP2C in liver [57]. Hydroxytolbutamide is then oxidized into carboxytolbutamide by alcohol dehydrogenase and aldehyde dehydrogenase and, finally, is exported out of the liver and excreted through the urine [58]. The results of the present study clearly showed that, along with the increase in activity of CYP2C6/11 and other CYP isozymes by APE and andrographolide, the AUC_0–12 h_ of plasma tolbutamide was decreased (Figure 2). This finding suggests that the plasma tolbutamide disposition can be changed by APE and andrographolide.

In the face of accelerating tolbutamide metabolism, we speculated that the hypoglycemic effect of tolbutamide would be impaired by APE and andrographolide. Therefore, we studied high-fat diet-induced obese mice displaying hyperglycemia. The results of these studies showed that, although APE and andrographolide increased mouse hepatic CYP2C6/11 expression (Figure 6) and decreased blood tolbutamide concentrations, as noted in rats, the AUC_0–8 h_ of blood glucose in APE-treated and andrographolide-treated mice was even lower than that of control mice (Table 4). This unexpected finding indicates that the impact of APE and andrographolide on the blood glucose-lowering of tolbutamide is likely to be counteracted by unidentified mechanisms. Andrographolide has been reported to induce glucose transporter 4 expression in rat soleus muscle and improve blood glucose uptake in streptozotocin-treated rats [12]. This suggests that the hypoglycemic effect of APE and andrographolide (APE, 77 ± 9 mg/dL; andrographolide, 80 ± 7 mg/dL; control, 104 ± 18 mg/dL; *P* < 0.05) may counteract, at least in part, their action on accelerating tolbutamide metabolism.

In conclusion, we have shown that both APE and andrographolide upregulate the gene transcription and enzyme activity of CYP1A1/2, CYP2C6/11, and CYP3A1/2 by activating AhR and PXR binding activity to DNA. The increase in CYP2C activity by APE and andrographolide leads to the accelerated metabolism of tolbutamide and a decrease in its disposition. APE and andrographolide, however, do not impair the hypoglycemic effect of tolbutamide.

## Figures and Tables

**Table 1:** Effects of ethanolic extract of *Andrographis paniculata* (APE) and andrographolide (AND) on the body weight, liver weight, and plasma aminotransferases of rats.

	Control	APE	AND
Body weight (g)			
Initial	271 ± 16	272 ± 7	272 ± 8
Final	337 ± 10^a^	307 ± 12^b^	332 ± 13^a^
Liver weight (g)	14.4 ± 1.2	13.3 ± 0.7	12.8 ± 1.5
Relative liver weight (%)	4.3 ± 0.4	4.3 ± 0.4	3.8 ± 0.3
Plasma aminotransferases (U/L)			
AST	24.8 ± 5.4	23.5 ± 5.7	27.7 ± 4.4
ALT	14.7 ± 2.4	12.9 ± 2.2	15.8 ± 8.2

Rats were orally dosed daily with 2 g/kg of APE or 50 mg/kg of AND for 5 days. Relative liver weight = (liver weight/ body weight) × 100. AST: aspartate aminotransferase; ALT: alanine aminotransferase. The values represent the mean ± SD, *n* = 5. The significance of the difference among group means was determined by one-way ANOVA followed by Tukey's test. ^ab^Values of treatments not sharing the same letter differ significantly, *P* < 0.05.

**Table 2:** Effects of ethanolic extract of *Andrographis paniculata* (APE) and andrographolide (AND) on the pharmacokinetic parameters of tolbutamide.

	Control	APE	AND
AUC_0–12 h_, (*μ*g/mL × h)	1393 ± 262^a^	881 ± 152^b^	1143 ± 123^ab^
*T*_max⁡_, h	1.6 ± 0.5	1.5 ± 0.6	1.0 ± 0.5
*C*_max⁡_, *μ*g/mL	151 ± 21	133 ± 21	140 ± 8
MRT, h	7.9 ± 1.6^a^	6.0 ± 1.5^b^	8.4 ± 1.1^a^

Rats were orally dosed daily with 2 g/kg of APE or 50 mg/kg of AND for 5 days. On day 6, a 20 mg/kg oral dose of tolbutamide was administered. AUC: area under the plasma drug concentration curve; *C*_max⁡_: the maximum plasma drug concentration; *T*_max⁡_: time to achieve *C*_max⁡_; MRT: mean resident time. The values represent the mean ± SD, *n* = 5. The significance of the difference among group means was determined by one-way ANOVA followed by Tukey's test. ^ab^Values of treatments not sharing the same letter differ significantly, *P* < 0.05.

**Table 3:** Effects of ethanolic extract of *Andrographis paniculata* (APE) and andrographolide (AND) on hepatic CYP450 and Phase II enzyme activities.

	Control	APE	AND
Cytochrome P450 content (pmol/mg protein)	486 ± 106^a^	680 ± 71^b^	674 ± 64^b^
Cytochrome *b5 *content (pmol/mg protein)	220 ± 62	289 ± 117	327 ± 79
NADPH-CYP450 reductase (*μ*mol/min/mg protein)	45.4 ± 3.1	56.2 ± 17.6	50.6 ± 7.1
Cytochrome P450s			
Ethoxyresorufin *O*-deethylase (1A1)	22.1 ± 6.7^a^	66.6 ± 25.3^b^	66.6 ± 13.5^b^
Methoxyresorufin *O*-demethylase (1A2)	13.0 ± 2.2^a^	23.8 ± 4.5^b^	25.6 ± 7.2^b^
Diclofenac 4-hydroxylase (2C)	15.0 ± 4.1^a^	45.1 ± 13.0^b^	43.8 ± 10.5^b^
Dextromethorphan *O*-deethylase (2D)	56.8 ± 12.7	85.3 ± 23.0	79.1 ± 30.4
* p-*Nitrophenol 6-hydroxylase (2E1)	47.2 ± 10.4^a^	88.9 ± 29.9^b^	91.1 ± 13.0^b^
Testosterone 6*β*-hydroxylase (3A)	513 ± 188^a^	831 ± 243^b^	1195 ± 284^b^
Midazolam 1-hydroxylase (3A)	233 ± 55^a^	343 ± 43^b^	320 ± 84^ab^
Lauric acid 12-hydroxylase (4A)	139 ± 27	162 ± 86	214 ± 88
Phase II enzymes			
UDP-glucuronosyltransferase	11.8 ± 3.9^a^	35.6 ± 12.5^b^	21.1 ± 6.5^a^
Glutathione *S*-transferase	627 ± 65	744 ± 142	634 ± 62

Rats were orally dosed daily with 2 g/kg of APE or 50 mg/kg of AND for 5 days. The activities of CYP isozymes and Phase II enzymes are expressed as pmol/min/mg protein and nmol/min/mg protein, respectively. The values represent the mean ± SD, *n* = 5. The significance of the difference among group means was determined by one-way ANOVA followed by Tukey's test. ^ab^Values of treatments not sharing the same letter differ significantly, *P* < 0.05.

**Table 4:** Effects of ethanolic extract of *Andrographis paniculata* (APE) and andrographolide (AND) on glucose tolerance test results in obese mice.

Plasma glucose (mg/dL)							
Time (h)	0	0.25	0.5	1	2	4	8
Control (*n* = 6)	104 ± 18^b^	225 ± 66^b^	164 ± 44^b^	111 ± 18	105 ± 27	105 ± 29^b^	105 ± 10^b^
APE (*n* = 6)	77 ± 9^a^	155 ± 26^a^	115 ± 24^a^	87 ± 25	87 ± 16	98 ± 13^b^	103 ± 11^b^
AND (*n* = 8)	80 ± 7^a^	152 ± 37^a^	124 ± 28^ab^	99 ± 29	88 ± 28	76 ± 17^a^	80 ± 17^a^

High-fat diet-induced obese mice were orally dosed daily with 10 mL/kg of 0.5% aqueous methyl cellulose, 2 g/kg of APE, or 50 mg/kg of AND for 5 consecutive days. After the 5th daily dose, the animals were deprived of food overnight. On day 6, a 20 mg/kg dose of tolbutamide was orally administered to each animal. Thirty minutes later, a 1 g/kg oral dose of glucose, as a solution in water, was administered to each mouse. Serial blood samples up to 8 h after the glucose dose were collected from each mouse, and plasma glucose concentrations were determined. The values represent the mean ± SD, *n* = 5. The significance of the difference among group means was determined by one-way ANOVA followed by Tukey's test. ^ab^Values of treatments not sharing the same letter differ significantly, *P* < 0.05.
